# Modeling MyD88 Deficiency *In Vitro* Provides New Insights in Its Function

**DOI:** 10.3389/fimmu.2020.608802

**Published:** 2020-12-23

**Authors:** Nils Craig-Mueller, Ruba Hammad, Roland Elling, Jamal Alzubi, Barbara Timm, Julia Kolter, Nele Knelangen, Christien Bednarski, Birgitta Gläser, Sandra Ammann, Zoltán Ivics, Judith Fischer, Carsten Speckmann, Klaus Schwarz, Nico Lachmann, Stephan Ehl, Thomas Moritz, Philipp Henneke, Toni Cathomen

**Affiliations:** ^1^ Institute for Transfusion Medicine and Gene Therapy, Medical Center–University of Freiburg, Freiburg, Germany; ^2^ Center for Chronic Immunodeficiency (CCI), Medical Center–University of Freiburg, Freiburg, Germany; ^3^ MD Program, Faculty of Medicine, University of Freiburg, Freiburg, Germany; ^4^ PhD Program, Faculty of Biology, University of Freiburg, Freiburg, Germany; ^5^ Institute for Immunodeficiency, Medical Center–University of Freiburg, Freiburg, Germany; ^6^ Center for Pediatrics and Adolescent Medicine, Medical Center–University of Freiburg, Freiburg, Germany; ^7^ Institute of Human Genetics, Medical Center–University of Freiburg, Freiburg, Germany; ^8^ Division of Medical Biotechnology, Paul-Ehrlich Institute, Langen, Germany; ^9^ Faculty of Medicine, University of Freiburg, Freiburg, Germany; ^10^ Institute for Clinical Transfusion Medicine and Immunogenetics Ulm, German Red Cross Blood Service Baden-Württemberg–Hessen, and Institute for Transfusion Medicine, University of Ulm, Ulm, Germany; ^11^ Institute of Experimental Hematology, Hannover Medical School, Hannover, Germany; ^12^ REBIRTH Cluster for Regenerative and Translational Medicine, Hannover, Germany

**Keywords:** MyD88, IRAK4, iMac, induced pluripotent stem cells (iPSC), transposon, cell therapy, gene therapy, Toll-like receptor

## Abstract

Inherited defects in MyD88 and IRAK4, two regulators in Toll-like receptor (TLR) signaling, are clinically highly relevant, but still incompletely understood. MyD88- and IRAK4-deficient patients are exceedingly susceptible to a narrow spectrum of pathogens, with ∼50% lethality in the first years of life. To better understand the underlying molecular and cellular characteristics that determine disease progression, we aimed at modeling the cellular response to pathogens *in vitro*. To this end, we determined the immunophenotype of monocytes and macrophages derived from MyD88- and IRAK4-deficient patients. We recognized that macrophages derived from both patients were particularly poorly activated by streptococci, indicating that both signaling intermediates are essential for the immune response to facultative pathogens. To characterize this defect in more detail, we generated induced pluripotent stem cells (iPSCs) of fibroblasts derived from an MyD88-deficient patient. The underlying genetic defect was corrected using *Sleeping Beauty* transposon vectors encoding either the long (L) or the short (S) *MYD88* isoform, respectively. Macrophages derived from these iPSC lines (iMacs) expressed typical macrophage markers, stably produced either MyD88 isoform, and showed robust phagocytic activity. Notably, iMacs expressing MyD88-L, but not MyD88-S, exhibited similar responses to external stimuli, including cytokine release patterns, as compared to genetically normal iMacs. Thus, the two MyD88 isoforms assume distinct functions in signaling. In conclusion, iPSC technology, in combination with efficient myeloid differentiation protocols, provides a valuable and inexhaustible source of macrophages, which can be used for disease modeling. Moreover, iPSC-derived macrophages may eventually aid in stabilizing MyD88-deficient patients during pyogenic infections.

## Introduction

Autosomal recessive mutations in the kinase IRAK4 (interleukin receptor-associated kinase 4) and the adaptor protein MyD88 (myeloid differentiation primary response 88) greatly impair Toll-like receptor (TLR) signaling. The resulting primary immunodeficiency is characterized by a substantial risk for life-threating pyogenic bacterial infections. MyD88 and IRAK4 deficiencies share clinically relevant characteristics that are biologically not well understood. First, the spectrum of pathogens to which MyD88- and IRAK4-deficient patients are susceptible is surprisingly narrow, with *Streptococcus pneumoniae*, *Staphylococcus aureus* and *Pseudomonas aeruginosa* accounting for >85% of invasive infections. To the contrary, mice lacking MyD88 show enhanced susceptibility to more than 20 bacterial species, several parasites and viruses (1). These differences between mouse and human remain largely elusive, yet they suggest redundancies in the human innate immune system that are not found in mice. Second, whereas most patients experience life-threatening infections in the first years of life (∼50% lethality), infection susceptibility ceases thereafter. This is illustrated by the fact that invasive bacterial infections have not been reported in patients above 14 years of age (2). Aberrations in mononuclear phagocytes are widely viewed to underlie infection susceptibility. However, the response of MyD88-deficient monocytes to signature pathogens is variable, and the monocyte response *per se* does not correlate well with the individual risk for infection at the time of testing. Moreover, whole blood transcriptomic studies of MyD88- and IRAK4-deficient patients showed a residual inflammatory response of patient cells to whole bacteria, while the response to purified TLR agonists was fully abrogated (3). All in all, these data emphasize our incomplete understanding of TLR signaling in human myeloid cells, which is hampered by the rarity of MyD88 and IRAK4 patients. Thus, a patient-specific myeloid model system of these deficiencies would be of substantial value not only for understanding the cellular basis of the deficiency but also for MyD88-mediated signaling more generally.

Following binding of their respective ligand, TLR dimerization allows for their respective intracellular Toll/IL-R1 (TIR) domains to interact. This in turn enables the binding of downstream TIR-possessing proteins, such as MyD88 (4). MyD88 is an essential adapter protein in TLR-signaling, since all TLRs - except for TLR3 - use MyD88 at least partially for signal transduction. The interactions between TIR domains are believed to be weak. Hence, the binding of multiple TIR-containing proteins, likely in positive cooperative binding or allostery, is required to activate a switch-like signal transduction process (4). Further, MyD88 contains a death domain (DD), which allows for the binding of other DD containing proteins, including members of the IRAK family. At least three different IRAK proteins bind to MyD88, namely IRAK 1, 2, and 4. Together, they form a higher-order structure called the Myddosome, in which up to eight MyD88 molecules form a complex with four IRAK 4 molecules. This structure is essential for proper signal transduction, ultimately activating nuclear factor kappa-light-chain-enhancer of activated B cells (NF-κB). There are two functionally distinct MyD88 isoforms. Full-length MyD88 (hereafter referred to as MyD88-L) mediates NF-κB activation. In contrast, a shorter splice variant lacking the intermediate domain (MyD88-S) is unable to activate NF-κB, but has been argued to function in a dominant negative manner (5–7). However, most experiments to evaluate the function of MyD88 or its two isoforms used transient expression in immortalized non-immune cells lines, such as HEK293 cells. In this context, it was shown that forced MyD88 expression causes NF-κB activation even in the absence of a ligand (8). These studies highlight the importance of controlling MyD88 expression levels, as the stoichiometry of MyD88 signaling is critically important when assessing physiological activation of NF-κB (4, 8).

While patient-derived primary immune cells are well suited to study the underlying cellular pathophysiology of disorders of the immune system, the patients are rare and the cells usually very limited in number. Induced pluripotent stem cells (iPSCs), on the other hand, have been shown to be valuable for both modeling and studying disease phenotypes *in vitro* and as a starting material to manufacture cell therapies (9–11). In the context of primary immunodeficiencies, iPSCs represent an unlimited source of patient-derived cells that can be differentiated into many immune cell types of the body (12), including B cells (13), T lymphocytes (14–17), NK cells (16, 18), and monocytes/macrophages (19–21). Because iPSCs can be transfected and subcloned, they are also well suited for genetic engineering, including stable gene transfer with the *Sleeping Beauty* (SB) transposon platform (22, 23). Unlike most viral gene transfer platforms, SB vectors integrate in a pseudorandom manner (‘TA’ sequences). Moreover, titration of the vector components allows for insertion of transgenes in copy numbers that enable transgene expression at physiological levels (24).

As MyD88 acts as an integrator of information, we aimed at establishing physiologically relevant cellular disease models in order to study the role of MyD88 on differentiation and inflammatory function of myeloid cells in general, and the function of the MyD88-L and MyD88-S isoforms in particular. We demonstrate with patient-derived cells that the inflammatory response to bacteria depends on both a functional MyD88 and the differentiation status of myeloid cells. Moreover, macrophages derived from iPSCs harboring MyD88-L—but not MyD88-S—exhibited comparable cellular response to external stimuli, including cytokine release patterns, as normal macrophages. This suggests that the two MyD88 isoforms assume distinct functions in signaling. In conclusion, patient-derived monocytes and iPSC technology combined with myeloid differentiation protocols represents a valuable source for *in vitro* disease modeling to study MyD88 deficiency. Furthermore, the genetically corrected derivatives of patient-derived iPSCs represent an unlimited source of autologous monocytes and macrophages that may be used as a future therapeutic option for stabilizing patients with acute bacterial infections.

## Materials and Methods

### Sleeping Beauty Transposon Vectors

The SB transposon vectors were generated using standard molecular cloning. The amino acid sequences of MyD88-S and MyD88-L MyD88 correspond to NP_001166039.1 and AAC50954.1, respectively. The SB100X transposase was previously described (23).

### Cell Culture

For all experiments, cell numbers and viabilities were determined by NucleoCounter NC-250 (ChemoMetec). HEK293T cells were cultured in DMEM high glucose GlutaMax medium (Thermo Fisher Scientific) supplemented with 10% FCS (PAA) and 1% penicillin/streptomycin (Sigma-Aldrich). HEK293T cells were transfected using polyethyleninine (PEI, Polysciences) as previously described (25).

### Induced Pluripotent Stem Cells

For generation of induced pluripotent stem cells (iPSCs), 50,000 fibroblasts of a MyD88-deficient patient were reprogrammed using Cytotune iPS 2.0 Sendai Reprogramming kit (Thermo Fisher Scientific) according to the manufacturer’s instruction. Emerging iPSC colonies were expanded in DMEM F/12 medium supplemented with 20% Knockout Serum Replacement (Thermo Fisher Scientific), 1% non-essential amino acids (Thermo Fisher Scientific), 1% l-glutamine (Biochrom), 1% penicillin/streptomycin (Sigma-Aldrich), 100 µM β-mercaptoethanol (Sigma-Aldrich), and 40 ng/ml of bFGF (Immunotools), on irradiated mouse embryonic fibroblasts (GlobalStem) as feeder cells. Alternatively, iPSCs were cultured under feeder-free conditions in mTeSR Basal Medium (Stem Cell Technologies) plus 1% penicillin/streptomycin on Matrigel-coated plates (Corning). For karyotyping and array-CGH (comparative genome hybridization; Agilent SurePrint G3 Human CGH Microarray 4x180K), iPSCs were grown under feeder-free conditions before being processed for karyotyping or genomic DNA extracted for array CGH using standard protocols.

### Generation of Transgenic iPSC Lines and Derivation of iMacs

Two million iPSCs in 100 µl of nucleofection mix (Mouse ES kit, Lonza) were mixed with plasmid DNA (3,75 µg of transposon DNA plus 1,25 µg of SB100X transposase expression vector) and nucleofected using Nucleofector 1D (program A023, Lonza). Seven days after nucleofection, puromycin selection was started. Transgene expression positive iPSC clones were identified using RT-PCR. Fully characterized iPSCs were differentiated to iMacs using previously established protocols (21). Briefly, iPSC colonies were harvested using dispase II (Roche) and concentrated by gravity. Embryonic body (EB) formation was induced by transfer of the iPSC colonies to EB-formation medium [DMEM/F-12 + Glutamax (LifeTechnologies), 20% KnockOut serum replacement (Gibco), 1% non-essential amino acids (LifeTechnologies), 1% penicillin/streptomycin (Sigma-Aldrich), 50 µM ß-mercaptoethanol (Sigma-Aldrich), 10 ng/ml of bFGF (Immunotools), and Rock inhibitor (Wako)] and placed in six-well suspension plates on an orbital shaker (100 rpm). Approximately 30 EBs were picked based on their large, dark, and relatively symmetric morphologies and plated onto gelatin-coated six-well adherent plates using macrophage differentiation medium I [X-vivo15 (Lonza), 1% penicillin/streptomycin, 1% glutamine, 50 ng/ml of hM-CSF (Immunotools), and 25 ng/ml of hIL-3 (Immunotools)]. Harvesting of iMacs started after ~3 weeks by collecting non-attached cells. The iMacs containing supernatant were passed through a 70-µm filter and centrifuged at 1000 rpm for 5 min, and cells were plated onto an adherent plate with macrophage differentiation medium II (macrophage differentiation medium I without hIL-3).

### MyD88 Expression Analyses

For RNA expression analysis, RNA was isolated from HEK293T cells or iPSCs using RNeasy Mini kit (Qiagen), and cDNA generated using QuantiTect Reverse Transcription kit (Qiagen) using primers #2606 5’-aactcatcgagaagaggtgtaggcg and #2607 5’-ccttgtccaaaaccatgatttggtgc. MyD88 amplicons were amplified by PCR using Phire Hot Start II polymerase (Thermo Fisher Scientific). For protein analysis, immunoblotting was performed as previously described (26) using an anti-MyD88 antibody (Santa Cruz). For loading control, a rabbit anti-β-actin (Cell Signaling Technology) was used.

### NF-κB Reporter Assay

80,000 HEK293T cells/well were plated in 24-well plates and co-transfected with the SB MyD88 expression vectors (600 ng) and a NF-κB reporter plasmid (600 ng) containing four copies of an NF-κB transcriptional response element (5’-GGGAATTTCC), a minimal CMV promoter, and a tdTomato reporter gene. On day 2 after transfection, cells were analyzed by flow cytometry on an Accuri C6 flow cytometer (BD Bioscience) to determine the percentage of tdTomato-expressing cells.

### Characterization of Myeloid Cells by Flow Cytometry

Monocyte subsets were analyzed by gating for CD14 (PacificBlue; BD Biosciences) and CD16 (PerCPCy5.5; BD Biosciences) in order to distinguish the three subsets of classical/inflammatory (HLA-DR^+^CD16^-^CD14^+^), intermediate (HLA-DR^+^CD16^+^CD14^+^) and nonclassical (HLA-DR^+^CD16^++^CD14^-^) monocytes, as described earlier (27). The gating strategy is depicted in **Figure S1F**. Sorting of monocyte subsets was performed on a MoFlo Astrios EQ (Beckman Coulter) cell sorter. To characterize iMacs, cells were stained for CD206 (APC, eBioscience), CD45 (PE Beckman-Coulter), CD33 (PerCP5.5, Biolegend), CD14 (FITC, Beckman-Coulter), CD11b (PE BD), or with the corresponding isotype controls and analyzed on an Accuri C6 flow cytometer (BD Bioscience).

### Phagocytosis Assays

50,000 iMacs were incubated with 1 million 2-µm red-labeled beads (Sigma-Aldrich). Cytochalasine b (10 µg/ml, Sigma-Aldrich) was added 6 h prior to addition of beads, where indicated. After 4 h, cells were analyzed on an Accuri C6 flow cytometer (BD Biosciences). Analysis of phagocytosis of human neutrophils was performed as described previously (28). Briefly, heat-killed *S. aureus* bacteria (Newman strain) were labeled with Alexa Flour 488 and co-incubated with neutrophil granulocytes isolated through Ficoll density gradient centrifugation. *S. aureus* were added for 1 or 15 min and fixed with 4% paraformaldehyde. Extracellular fluorescence was quenched with trypan blue to exclude fluorescence emitted from incompletely phagocytosed bacteria.

### Cytokine Release Assay

10^5^ PBMCs, isolated from EDTA blood using Ficoll density centrifugation, were stimulated with lipopolysaccharide (LPS at 10 or 100 ng/µl), flagellin (2.5 µg/ml) or heat-fixed bacteria (*S. aureus* or *Streptococcus agalactiae*, 10^7^/ml) for 20 h. IL-6 concentration in the supernatant was determined by amplified luminescent proximity homogeneous assay (AlphaLISA) according to manufacturer’s instructions (PerkinElmer). Intracellular staining for TNF was performed as described recently by flow cytometry (TNF-PE, BD) (27). To analyze monocyte-derived macrophages, PBMCs were differentiated for 7 d with 50 ng/ml of hMCSF (Immunotools). To measure TNF expression, sorted monocyte subsets were seeded into 48-well-plates. After 1 h of settling, cells were stimulated with 100 ng/ml of LPS or 5 × 10^7^ CFU/ml of heat-fixed *S. agalactiae* for 2 h. RNA was extracted with the RNeasy Microkit (Qiagen), followed by RT-PCR with the SuperScript™ IV VILO cDNA synthesis kit (Invivogen), both according to manufacturer`s instructions. qRT-PCR was performed using Absolute SYBR Green (ThermoScientific) and a Realplex Masterycler (Eppendorf). Expression levels of TNF (primers 5’-CTCCCAGGTCCTCTTCAAGG and 5’-ATAGTCGGGCCGATTGATCT) were normalized to *GAPDH* (primers 5’-ACACCCACTCCTCCACCTTT and 5’-TACTCCTTGGAGGCCATGTG). Otherwise, 50,000 iMacs were stimulated with 5 million heat-inactivated *S. aureus* (Thermo Fisher Scientific). Supernatants were removed at specified time points and evaluated by Cytokine Bead Array (BD Biosciences) on a BD Canto II according to the manufacture’s instruction. The mean fluorescent intensity (MFI) was used for comparative data analysis.

### Statistical Analysis

For cytokine release assay, at least 1,000 beads per cytokine for each experimental sample were dissected in triplicate. As the same beads are used in each experiment, the MFI was suitable for comparing samples. An unpaired two-tailed Student’s t test (GraphPad Prism 8, La Jolla, California) was applied for testing significant differences between samples (p < 0.05): Horizontal bar indicates average with standard deviation, and all other bar graphs show mean and standard deviation.

### Ethics

This study was approved by the ethics committee of the University of Freiburg.

## Results

### Disease Modeling With Patient-Derived Monocytes/Macrophages

**Figure 1:**
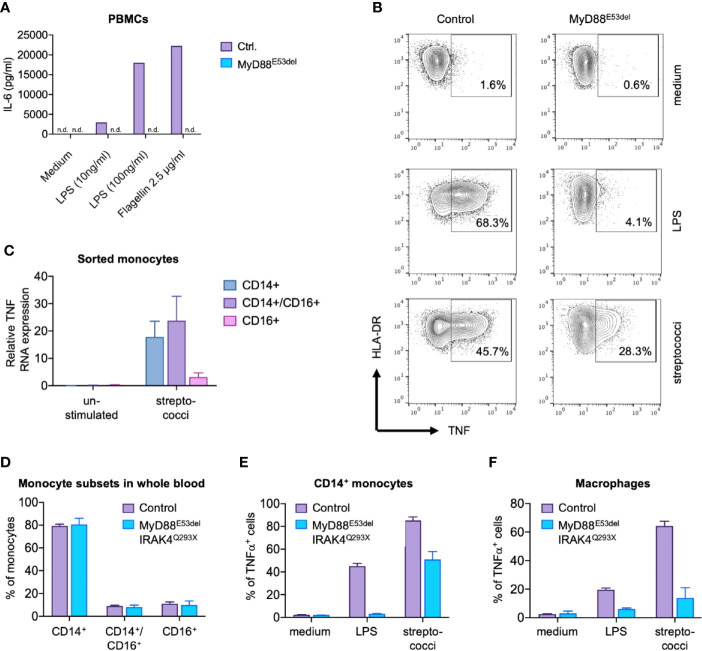
MyD88-dependent and MyD88-independent signaling in MyD88 deficiency. **(A)** IL-6 secretion. Peripheral blood mononuclear cells (PBMCs) of a MyD88^E53del^ patient were stimulated with lipopolysaccharide (LPS) or flagellin, and IL-6 concentration determined in supernatant. **(B)** TNF expression. Monocytes of a MyD88^E53del^ patient were stimulated with LPS or streptococci, and intracellular TNF expression determined by flow cytometry. **(C)** TNF expression in monocyte subsets. TNF expression was determined in sorted monocyte subsets of a healthy donor upon stimulation with streptococci. **(D)** Monocyte subsets in MyD88/IRAK-4 deficiency. Shown is the proportional subset distribution. **(E, F)** TNF expression in patient-derived monocytes **(E)** and macrophages **(F)**. Presented is the fraction of TNF-positive cells of healthy donors (control) or MyD88^E53del^/IRAK44^Q293X^ patients upon stimulation with LPS or streptococci.

To better understand the underlying molecular characteristics that determine disease progression, we first analyzed the basic immunophenotype of monocytes derived from MyD88 and IRAK4-deficient patients with distinct courses of disease. The index patient of a MyD88-deficient kindred (**Figure S1A**) II.2 suffered from multiple pyogenic infections in infancy, ultimately leading to genetic workup of a suspected TLR signaling defect. Sequencing of the MyD88 gene revealed a known pathogenic homozygous mutation (p.Glu53del, **Figure S1B**) (29). Despite antibiotic prophylaxis (immunoglobulin substitution therapy was refused by the parents), the patient experienced overall three streptococcal bloodstream infections and multiple soft tissue infections in the first 5 years of life. Yet, the patient fully recovered from all events, displayed normal psychomotor development, and has not experienced further invasive bacterial infections since the age of 9 years (**Figure S1C**). When II.3, a younger sibling of II.2, was born, antibiotic prophylaxis was started after the diagnosis of MyD88 deficiency at the age of 5 weeks. The patient received timely vaccinations, including three doses of 13-valent pneumococcal conjugate vaccine. Despite a high alert for infections, the infant died from fulminant pneumococcal sepsis (non-vaccine-type 23B) and meningitis at the age of 8 months due to cardiac arrest 12 h after the onset of fever (**Figure S1D**). Although abundant pneumococci were present in the cerebrospinal fluid, pleocytosis was only marginal (**Figure S1E**), most likely due to the failure of leukocyte recruitment to the central nervous system. TLR-4 and TLR-5 ligands, lipopolysaccharide (LPS) and flagellin, respectively, failed to induce cytokine expression in MyD88-deficient peripheral blood mononuclear cells (PBMCs; **Figure 1A**), whereas the response to whole bacteria was partially preserved (**Figure 1B**). Monocyte subsets were previously found to differ in the recognition of glycolipids and nucleic acids (30, 31), which are essential cytokine-inducing effectors of Gram-positive bacteria (32). Thus, we wondered whether the cytokine response to bacteria in MyD88/IRAK4-deficiency resulted from alterations in monocyte subsets. Accordingly, we determined which monocyte subsets mediated the TNF response to streptococci in healthy subjects (27). We found that sorted CD14^+^ classical and CD14^+^/CD16^+^ intermediate monocytes, but not CD14^dim^ monocytes, responded to streptococci with substantial TNF formation (**Figure 1C**). Notably, monocyte subsets were qualitatively normally distributed in both MyD88- and IRAK4-deficient patients. Thus, MyD88 and IRAK4-dependent signal transduction appears not to be required for the development of human monocyte subsets (**Figure 1D** and **Figure S1F**). We next addressed whether differentiated macrophages mount a MyD88-independent response. Whereas over 50% of IRAK4 and MyD88-deficient CD14+ monocytes synthesized TNF after stimulation with streptococci (**Figure 1E**), macrophages derived from these monocytes were largely devoid of this response (**Figure 1F**). These results imply that mature macrophages, reflective of those residing in barrier tissues, but not their putative progenitors, critically rely on TLR/MyD88 signaling for sensing streptococci. For this reason, we chose to focus on macrophages for rest of the study.

### Assessment of MyD88-L and MyD88-S Expression Vectors

**Figure 2:**
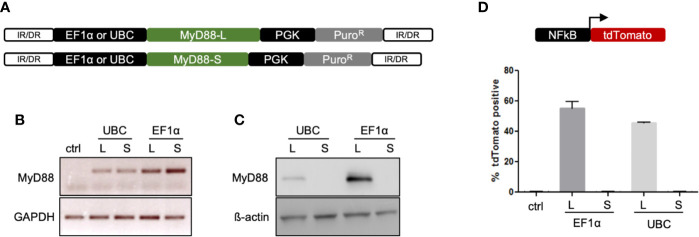
Sleeping Beauty vectors for genome correction. **(A)** Schematic of the Sleeping Beauty (SB) transposon system containing either the MyD88-L or MyD88-S transgene. Transposition leads to stable integration of the MyD88 transgene into the genome. **(B)** Expression of MyD88 RNA. RT-PCR was performed to detect MyD88 mRNA expression from SB vectors in transfected HEK293T cells. Used primers amplified an amplicon in exon 3, which is present in both MyD88-L and MyD88-S. **(C)** Expression of MyD88 protein. Western blot was performed to evaluate transgene expression from SB vectors in transfected HEK293T cells. **(D)** MyD88-dependent activation of NF-κB. HEK293T cells were co-transfected with a NF-κB reporter construct and SB MyD88 expression vectors. Activation of NF-κB was assayed by measuring the faction of tdTomato-positive cells. IR/DR, indirect/direct repeats; GAPDH, glycerinaldehyd-3-phosphat-dehydrogenase; UBC, ubiquitin C promoter; EF1α, elongation factor 1α promoter.

In order to correct the genetic defect, codon-optimized MyD88-L and MyD88-S splice variants were synthesized and cloned into a SB vector, either under control of an elongation factor 1α (EF1α) or the ubiquitin C (UBC) promoter, respectively (**Figure 2A**). To evaluate expression of MyD88-L or MyD88-S, HEK293T cells were transfected with the respective SB vectors. Reverse transcription on extracted RNA from these cells confirmed transcription of either MyD88 construct, with somewhat higher RNA expression levels from the EF1α driven constructs (**Figure 2B**). Protein expression from the transgene was detected by immunoblotting of cell lysates of the transfected HEK293T cells. MyD88-L, but not MyD88-S, was detected, suggesting that the used MyD88 antibody recognizes an epitope in the intermediate domain, which is missing in MyD88-S (**Figure 2C**). Western blot analysis further confirmed higher expression from the EF1α promoter. Overexpression of MyD88 is known to constitutively activate NF-κB even in the absence of IL-1R or TLR signaling (8). To assess functionality of the cloned MyD88 constructs, a reporter assay was conducted. HEK293T cells were co-transfected with the SB MyD88 expression vector and an NF-κB reporter plasmid harboring a tdTomato marker gene. Overexpression of MyD88-L, but not MyD88-S, activated the NF-κB reporter in HEK293T cells, suggesting that the intermediate domain is essential for activation of the NF-κB pathway (**Figure 2D**). In conclusion, the data confirmed expression of functional MyD88 from our SB constructs, confirming, as previously shown, that the MyD88 intermediate domain is necessary for activating NF-κB. Furthermore, the results underscore the importance of fine-tuning the expression levels of MyD88 in order to prevent signal transduction in the absence of TLR ligands.

### Generation of Patient-Derived Induced Pluripotent Stem Cells

**Figure 3:**
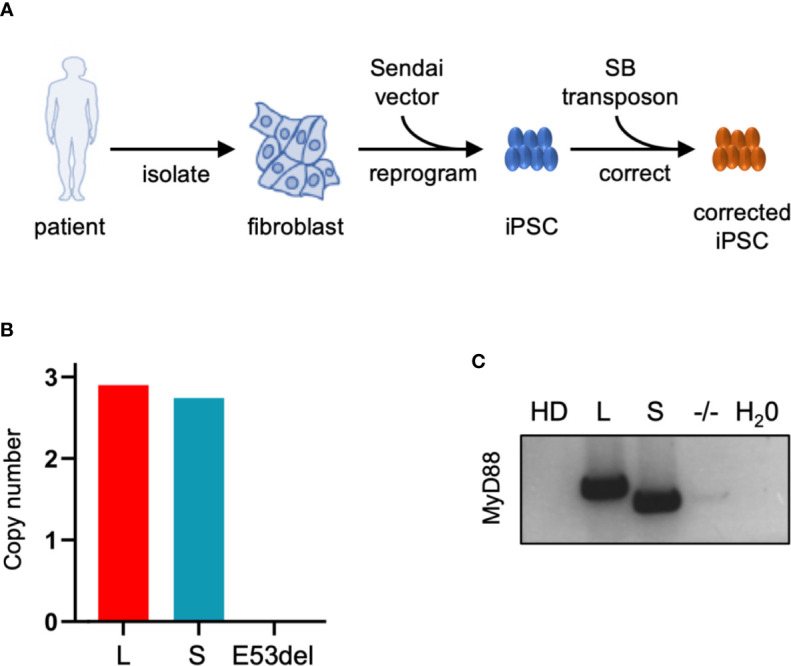
Generation of iPSCs derived from MyD88-deficient patient. **(A)** Schematic of iPSC generation and genetic correction with SB vectors. **(B)** SB copy number. Copy number of MyD88-L (L) and MyD88-S (S) transgene cassettes in iPSC clones was determined by digital droplet PCR. **(C)** MyD88 expression. RT-PCR was applied to assess MyD88 mRNA expression from SB vectors in MyD88-L and MyD88-S iPSC clones. SB, Sleeping Beauty vector; HD, healthy donor; E53del, non-corrected iPSC clone. Used primers amplified an amplicon that differentiates between MyD88-L and MyD88-S.

Fibroblasts of the afore-mentioned MyD88 patient (II.2) were isolated and reprogrammed to iPSCs using a Sendai virus-based system (**Figure 3A** and **Figure S2A**). Quality controls performed on iPSC clones (here shown for the clone used in further experiments) confirmed expression of pluripotency markers (**Figure S2B**), the presence of the underlying genetic mutation (ΔGAG, E53del; **Figure S2C**), an intact karyotype (**Figure S2D**), and the absence of gross genetic deletions/duplications (**Figure S2E**). This iPSC clone (E53del) was nucleofected with the EF1α driven MyD88-L and MyD88-S SB vectors and a SB transposase expression plasmid to stably integrate the SB vectors into the genome of the patient-derived iPSCs (**Figure S2A**). Puromycin was used to select for stably transfected iPSC clones, and post-nucleofection/post-selection clones were cultured for up to five months. A second round of quality controls on the generated iPSC clones employing karyotype analysis and array-comparative genome hybridization (array-CGH) detected no genetic aberrations following nucleofection, selection, and expansion of the MyD88-L and MyD88-S clones (data not shown). Two MyD88-L clones (L1 and L2) and one MyD88-S iPSC clone, along with the uncorrected MyD88^E53del^ (-/-) clone and an iPSC clone derived from a healthy donor (HD), were used for further experiments (**Figure S2A**). Using digital droplet PCR (ddPCR), the number of SB vector integration events was determined and revealed roughly three vector copies [MyD88-L, 2.9; MyD88-S, 2.8; MyD88^E53del^ (-/-), 0] in either of the iPSC clones (**Figure 3B**). Furthermore, RT-PCR analysis employing MyD88 transgene specific primers confirmed stable expression from the integrated SB vectors in the MyD88-L and MyD88-S iPSC clones (**Figure 3C**).

### Disease Modeling With Induced Pluripotent Stem Cells-Derived Macrophages

**Figure 4:**
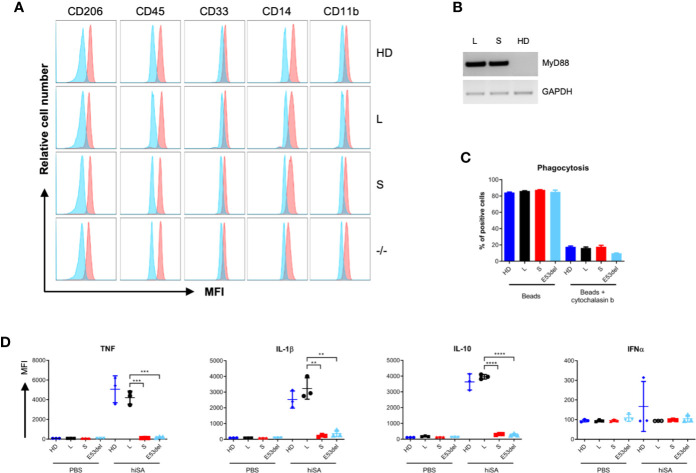
Functional correction of patient-derived iPSCs. **(A)** Surface marker analysis. Patient-derived iMacs were assessed by flow cytometry for expression of macrophage markers CD206, CD45, CD33, CD14, CD11b from the following clones: (HD), MyD88-L (L), MyD88-S (S), and MyD88^E53del^ (-/-) iPSC lines. In blue, isotype control. **(B)** MyD88 expression. RT-PCR was applied to assess MyD88 mRNA expression in differentiated iMacs derived from MyD88-L (L), MyD88-S (S), or healthy donor (HD) iPSC clones. Used primers amplified an amplicon that do not differentiate between MyD88-L and MyD88-S. **(C)** Phagocytosis activity. Flow cytometric analysis to verify uptake of red-fluorescent beads in the absence or presence of cytochalasin B. Displayed is the percentage of red-fluorescent iMacs. **(D)** Cytokine secretion. iMacs were exposed to heat-inactivated *S. aureus* (hiSA). The concentrations of secreted cytokines in the supernatant were determined by cytometric bead array. All data points in triplicate. *p*-values (unpaired two-tailed Student’s t test): *P < 0.05, **P < 0.01, ***P < 0.001, ****P < 0.0001. MFI, mean fluorescent intensity.

To validate MyD88-deficiency as the underlying molecular defect in sensing bacteria and in order to assess the ability of MyD88-L or MyD88-S isoforms to compensate for the genetic defect, iPSC clones MyD88-L, MyD88-S, the uncorrected MyD88^E53del^ (-/-) clone, along with iPSCs from an immunologically healthy donor (HD) were differentiated into macrophages (iMacs). SB-harboring iMacs looked morphologically like macrophages, displayed typical macrophage markers (**Figures 4A**) and stably expressed the MyD88 transgene upon myeloid differentiation (**Figure 4B**). To validate functionality of these iMacs, a phagocytosis assay with red fluorescent-labeled beads war performed. Flow cytometric analysis confirmed robust phagocytic activity for iMacs derived from all iPSC clones (33), which could be prevented by the actin filament polymerization inhibitor cytochalasin B (**Figure 4C**).

Because MyD88-deficient patients are highly susceptible to *S. aureus* (2, 34), we decided to evaluate the response of iMacs to this bacterial species. We hypothesized that cytokine production following exposure to heat-inactivated *S. aureus* (hiSA) would be highly dependent on MyD88-mediated signaling (34). iMacs derived from the MyD88-L iPSC clone secreted proinflammatory cytokines (TNF, IL-1ß, and IL-10) to a similar extent as iMacs derived from normal iPSC. In contrast, MyD88-S expressing iMacs and uncorrected MyD88-deficient (MyD88^E53del^) iMacs did not respond to the stimuli (**Figure 4D**). In contrast, IFNα was not secreted by iMacs following stimulation with *S. aureus* in general. Due to the pseudorandom nature of SB integration, we validated these results with a second MyD88-L clone. The results obtained with clone MyD88-L1 basically mirror the data presented for iPSC clone MyD88-L2 (**Figures S3B–G**). Unexpectedly, however, IL-8 secretion was significantly higher in MyD88-S expressing and MyD88 uncorrected iMacs as compared to macrophages derived from iPSC clones MyD88-L1 and MyD88-L2 (**Figure S3G** and **S4**); these data also show that MyD88-S and MyD88^E53del^ iMacs are still capable of cytokine production following stimulation with heat-inactivated *S. aureus* in an MyD88-independent manner. Interestingly, we did not detect any differences between uncorrected and MyD88-S expressing clones, suggesting that the short MyD88 splice variant does not act in a dominant-negative fashion in our model. In summary, patient-derived iPSCs could be genetically corrected by stable integration of a MyD88-L expression cassette. Macrophages derived from these corrected iPSCs revealed were comparable cellular immune response to normal iMacs, which supports that our model may serve as a valid and physiologically relevant paradigm to study MyD88 deficiency in particular, and TLR signaling defects in general.

## Discussion

In our study we compared TLR signaling outputs of monocytes and monocyte-derived macrophages derived from a MyD88- and an IRAK4-deficient patient in response to purified TLR ligands and whole bacteria. We found that the NF-κB response was fully abrogated in both monocytes and macrophages lacking MyD88/IRAK4 when exposed to purified TLP ligands, such as LPS. On the other hand, the relevant residual inflammatory response of monocytes when exposed to whole bacteria suggests a MyD88- and IRAK4-independent but NF-κB-dependent inflammatory response. In contrast, we found MyD88/IRAK4-deficient macrophages to almost fully depend on an intact TLR–MyD88/IRAK4–NF-κB axis for their response to whole bacteria. A similar phenotype has been observed in transcriptomic analyses of whole blood samples from MyD88 and IRAK4 deficient subjects stimulated with TLR ligands and whole bacteria, where the immune cell specific response was not resolved (3).

Following up on the results from primary patient-derived monocytes/macrophages, a disease model based on iPSC-derived macrophages was established. First, we corrected the genetic lesion in the MyD88 patient-derived iPSCs using the SB transposon system. MyD88 transgene expression remained stable over several months of iPSC culture, consistent with very low levels of transcriptional silencing of SB vector insertions in human cell lines (35) and in transgenic animals (36). Importantly, differentiation to iMacs did not induce transgene silencing either, a problem that has been described for other iPSC differentiation protocols [e.g. (37)]. Our *in vitro* disease model of MyD88 deficiency revealed a dramatic decrease in the ability of uncorrected iMacs to secrete proinflammatory cytokines following stimulation with heat-inactivated *S. aureus*. On the other hand, and in accordance with the analysis of patient-derived primary cells, MyD88 was not essential for differentiation into iMacs. The defect in cytokine production was rescued by the transfer of a canonical full-length MyD88 transgene cassette, but not with a shorter MyD88-S splice variant. Unexpectedly however, we observed an increase in IL-8 secretion of uncorrected and MyD88-S expressing iMacs following stimulation. This implies that MyD88 deficiency does not simply lead to a general decrease in cytokine production but perhaps alters the cytokine profile. Other groups have shown that there are MyD88-independent mechanisms for activating IL-8 secretion (38). On the other hand, we find the short MyD88 splice variant not to act, at least in our model, in a dominant-negative fashion, as has been concluded previously using transient overexpression of MyD88-S in other models (7). MyD88 signaling seems very sensitive to the concentration of signaling molecules. Thus, plasmid-based overexpression may result in protein concentrations orders of magnitude greater than the physiological level (39). In conclusion, the iMac model system may allow for a more physiological dissection of MyD88-related immune defects.

It is tempting to speculate that invasive bacterial disease episodes of MyD88/IRAK4-deficient patients, which are believed to often arise endogenously through epithelial or mucosal surfaces, originate from the severe signaling defect in tissue macrophages, as they are unable to contain bacteria at epithelial or mucosal surfaces. In contrast, monocytes circulating in the blood seem to mount a residual inflammatory response. From a clinical perspective, the fulminant fatal pneumococcal sepsis of the described MyD88-deficient infant illustrates the limited therapeutic options, once a bloodstream infection in the context of a TLR signaling defect occurs. Given the strongly improved prognosis of MyD88/IRAK4 deficiency after adolescence, hematopoietic stem cell transplantation is currently not considered as a first-line treatment for these patients (2). Our data suggest that the SB transposon system can be used to genetically modify iPSCs with subsequent differentiation into iMacs without gene silencing. As the genetically corrected iMacs behaved similar to those derived from a healthy donor, autologous gene-corrected macrophages, or their precursors may represent a viable cell therapeutic option to stabilize MyD88-deficient patients in emergency situations.

In summary, a combination of data from primary patient-derived myeloid cells and iPSC-derived iMacs shows that MyD88-deficiency in humans does neither affect cell differentiation nor their non-TLR signaling related functions. MyD88-deficiency does, however, dramatically affect the cytokine response following stimulation with the clinically relevant staphylococci and streptococci. Moreover, small differences in the stoichiometry of signaling molecules can substantially impact on downstream signaling. Our iPSC-based cellular disease model might therefore allow for the detection of subtle and physiologically relevant differences, when studying MyD88 deficiency in particular, and TLR signaling defects in general.

## Data Availability Statement

The original contributions presented in the study are included in the article/**Supplementary Material**; further inquiries can be directed to the corresponding author/s.

## Author Contributions

TC and PH conceptualized the study. NM, RH, RE, JA, ZI, JF, CS, KS, NL, SE, TM, PH, and TC developed and designed the methods. NM, RH, RE, JA, BT, JK, NK, CB, BG, and SA acquired the data. NM, RH, RE, JA, BG, PH, and TC analyzed the data. All authors discussed the data. NM, RH, RE, PH, and TC wrote the manuscript. JA, PH, and TC supervised the study. TC, PH, NL, TM, and SE acquired the funding. All authors contributed to the article and approved the submitted version.

## Funding

This work was supported by the German Federal Ministry of Education and Research (iMacNet–01EK1602 to TC, PH, NL, and TM, 01GL1746A to PH, and IFB–01EO1303 to TC, PH, and SE), the Else Kröner-Fresenius Foundation (to PH), the German Research Foundation (HE3127/9, HE3127/12, and SFB/TRR167 to PH), and the Research Commission of the Faculty of Medicine of the Albert-Ludwigs-University of Freiburg (3096997002 to TC). The article processing charge was funded by the Baden-Wuerttemberg Ministry of Science, Research and Art and the University of Freiburg in the funding program Open Access Publishing.

## Conflict of Interest

The authors declare that the research was conducted in the absence of any commercial or financial relationships that could be construed as a potential conflict of interest.
