# Supplementary material for: Modeling MyD88 Deficiency In Vitro Provides New Insights in Its Function
Source: Front Immunol. 2020 Dec 23;11:608802. doi: 10.3389/fimmu.2020.608802 (PMC7786022; doi:10.3389/fimmu.2020.608802)
Supplement: Supplementary file 1 [file DataSheet_1.pdf]

SUPPLEMENTARY MATERIAL

Modeling MyD88 deficiency *in vitro* provides new insights in its function

Nils Craig-Mueller[1,2,3,+], Ruba Hammad[1,2,4,+], Roland Elling[2,5,6,+], Jamal Alzubi[1,2], Barbara Timm[1,2], Julia Kolter[2,5], Nele Knelangen[2,5], Christien Bednarski[1,2], Birgitta Gläser[8], Sandra Ammann[2,5], Zoltán Ivics[7], Judith Fischer[8,9], Carsten Speckmann[2,6], Klaus Schwarz[10], Nico Lachmann[11,12], Stephan Ehl[2,5,9], Thomas Moritz[11,12], Philipp Henneke[2,5,6,9,*], Toni Cathomen[1,2,9,*]

[1] Institute for Transfusion Medicine and Gene Therapy, Medical Center – University of Freiburg, Freiburg, Germany

[2] Center for Chronic Immunodeficiency (CCI), Medical Center – University of Freiburg, Freiburg, Germany

[3] MD Program, Faculty of Medicine, University of Freiburg, Freiburg, Germany

[4] PhD Program, Faculty of Biology, University of Freiburg, Freiburg, Germany

[5] Institute for Immunodeficiency, Medical Center – University of Freiburg, Freiburg, Germany

[6] Center for Pediatrics and Adolescent Medicine, Medical Center – University of Freiburg, Freiburg, Germany

[7] Division of Medical Biotechnology, Paul-Ehrlich Institute, Langen, Germany

[8] Institute of Human Genetics, Medical Center – University of Freiburg, Freiburg, Germany

[9] Faculty of Medicine, University of Freiburg, Freiburg, Germany

[10] Institute for Clinical Transfusion Medicine and Immunogenetics Ulm, German Red Cross Blood Service Baden-Württemberg – Hessen, and Institute for Transfusion Medicine, University of Ulm, Ulm, Germany

[11] Institute of Experimental Hematology, Hannover Medical School, Hannover, Germany

[12] REBIRTH Cluster for Regenerative and Translational Medicine, Hannover, Germany

[+] these authors contributed equally

[*] these authors contributed equally

[*] Correspondence:

Toni Cathomen, toni.cathomen@uniklink-freiburg.de

Philipp Henneke, philipp.henneke@uniklink-freiburg.de

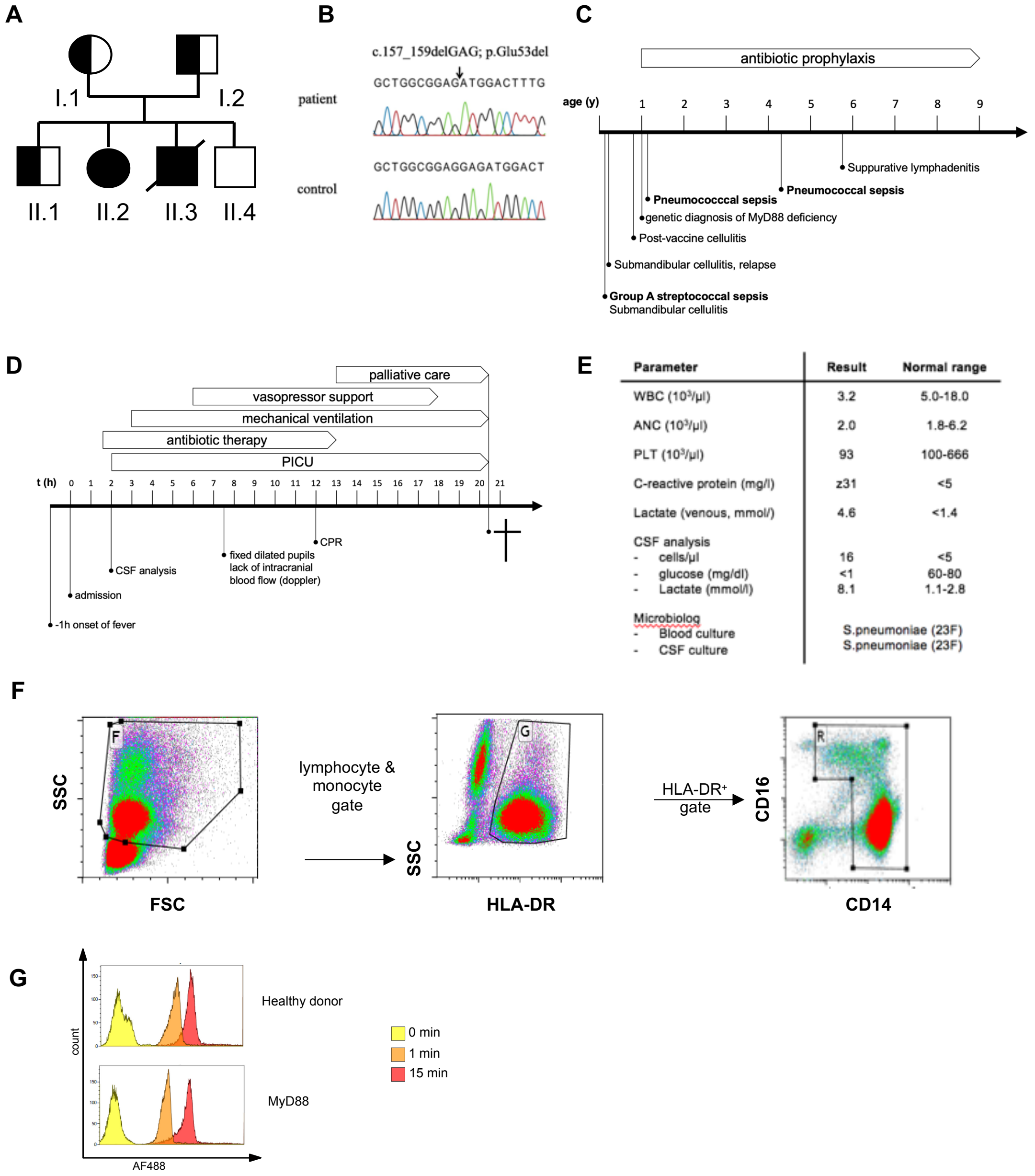

Figure S1. Clinical course of MyD88-deficient kindred. (**A**) Pedigree of the family with the index patient II.2 and the affected brother II.3. (**B**) Mutational analysis of MyD88 from patient II.2 illustrating deletion of E53. (**C**) Infectious disease history of patient II.2. (**D**) Clinical course of foudroyant pneumococcal sepsis of patient II.3. (**E**) Laboratory and microbiology results of patient II.3 at admission with fatal pneumococcal sepsis. (**F**) Gating strategy. **(G)** Phagocytosis by polymorphonuclear cells (PMNs) from index patient II.2 compared to PMNs from healthy donor.

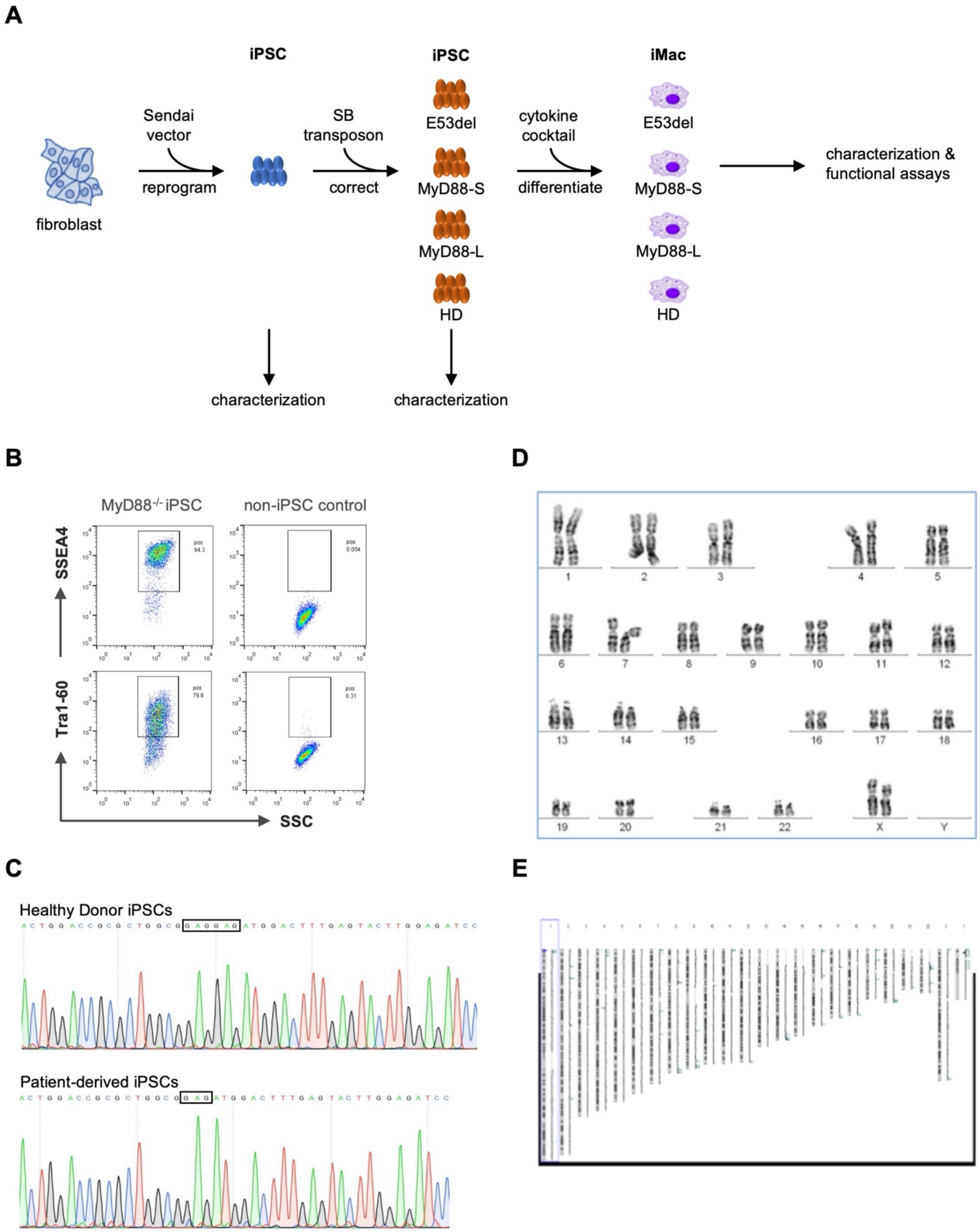

Figure S2. Experimental outline. (**A**) Experimental setup for production of the different patient-derived iMac lines. (**B**) Patient-derived iPSCs express pluripotency markers. (**C**) Sequencing of patient-derived iPSCs confirms E53del mutation. (**D**) Karyotype analysis and (**E**) array-Comparative Genome Hybridization shows no chromosomal abnormalities following iPSC-reprogramming. Exemplarily shown are data for the patient-derived E53del line. SB, Sleeping Beauty transposon; iMac, iPSC-derived macrophage; MyD88-S and MyD88-L, lines corrected with SB vector expressing short or long MyD88 variant, respectively; HD, healthy donor.

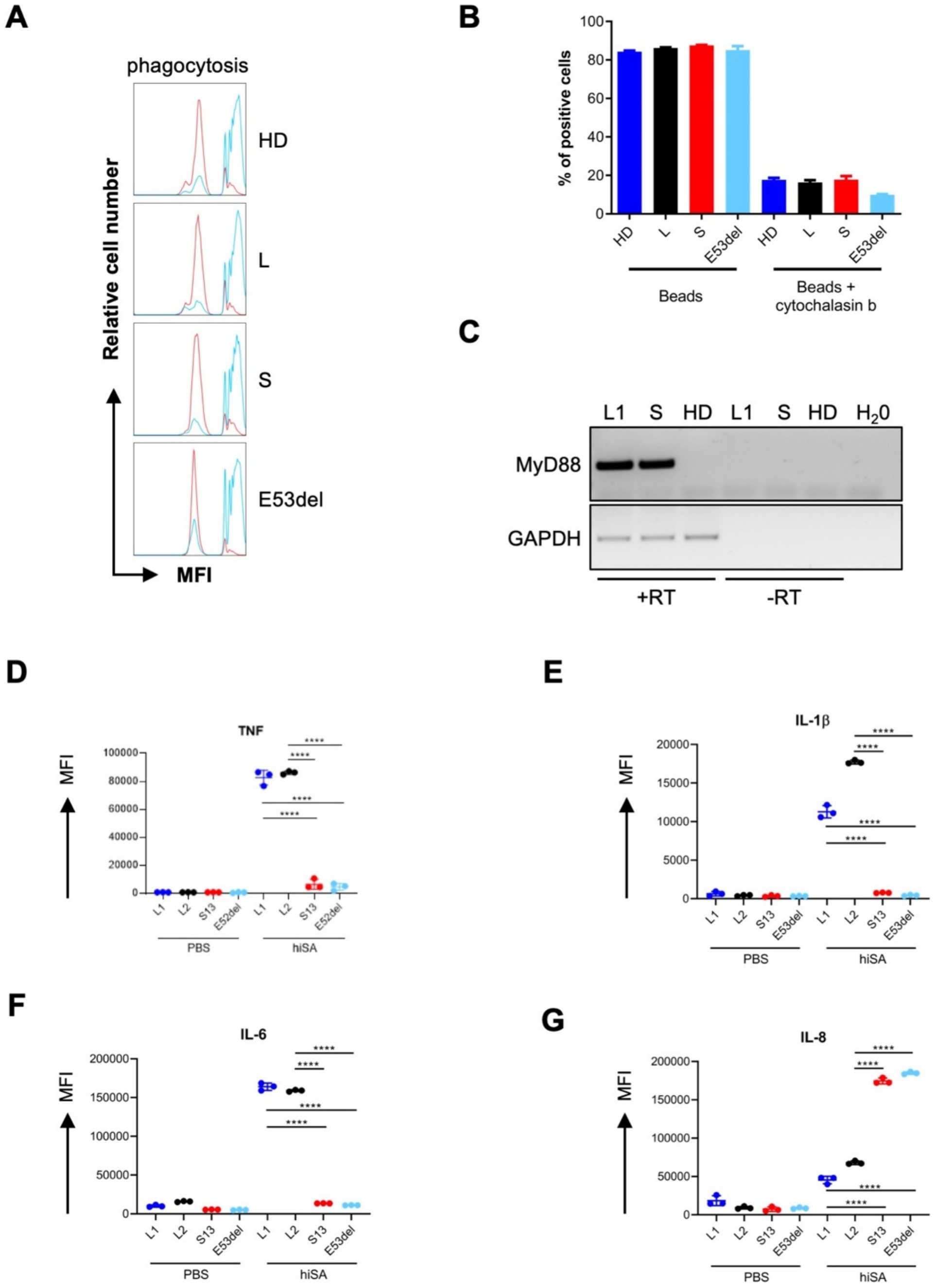

Figure S3. Characterization of iMacs. (**A**) Ability of phagocytosis by all iMac clones demonstrated by efficient uptake of fluorescently labeled beads. Shown are representative figures, with red curves displaying impaired phagocytosis following coincubation with cytochalasin B. **(B)** Summary of same phagocytosis experiment in triplicate (n=3). (**C**) Stable MyD88 transgene expression was demonstrated by RT-PCR in iMacs derived from iPSCs that were cultured for >6 months. (**D-G**) Comparable cytokine release profiles of iMacs derived from two different MyD88-L iPSC clones (clones L1, L2) after stimulation with heat-inactivated Staphylococcus aureus (hiSA). MFI, mean fluorescent intensity.

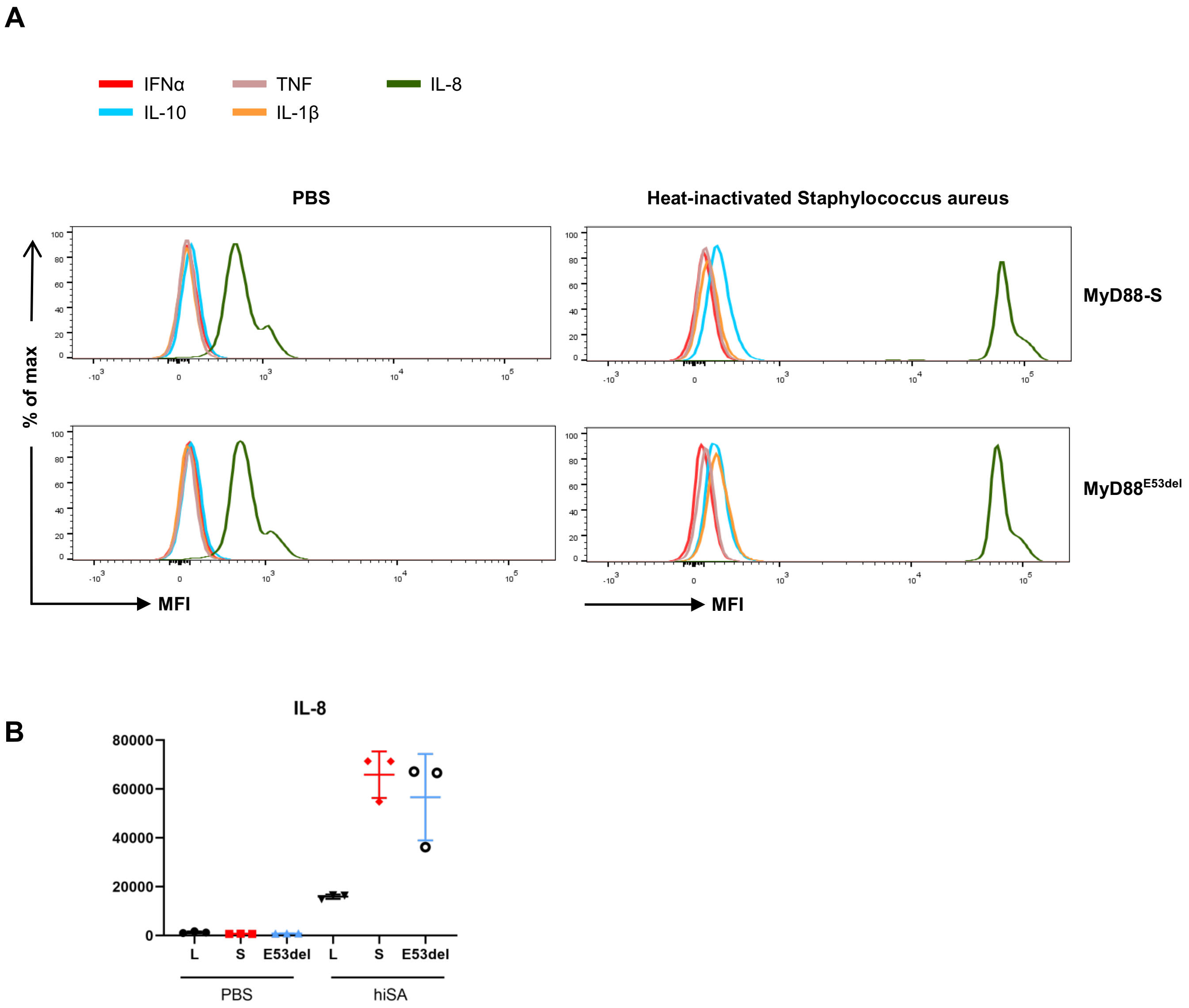

Figure S4. Cytokine release profiles of MyD88-S and MyD88^{E53del} macrophages show robust IL-8 production following stimulation with heat-inactivated Staphylococcus aureus (hiSA). (**A**) Representative cytometric bead array data. MFI (x-axis) is plotted against percentage of total beads (y-axis). Following stimulation, a shift for IL-8 (green) was observed. **(B)** Summary of cytometric bead array data. Shown is IL-8 production (n=3) from samples MyD88-L (L), MyD88-S (S), and MyD88^{E53del} (E53del).
